# Effects of UV-A/B/C on flavonoids and related synthetic enzymes in *Tetrastigma hemsleyanum*

**DOI:** 10.3389/fpls.2024.1477280

**Published:** 2024-10-23

**Authors:** Shan Li, Jingqing Xia, Shouzan Liu, Zhe Li, Qiong Shen, Feng Yang, Xinhong Liu, Yan Bai

**Affiliations:** ^1^ College of Food and Health, Zhejiang Agriculture and Forestry University, Hangzhou, China; ^2^ Zhejiang Provincial Key Laboratory of Resources Protection and Innovation of Traditional Chinese Medicine, Hangzhou, China; ^3^ Botanical Garden, Zhejiang Agricultural and Forestry University, Hangzhou, China; ^4^ Zhejiang Academy of Forestry, Hangzhou, China

**Keywords:** *Tetrastigma hemsleyanum*, UV, darkness treatment, flavonoids, enzyme activity

## Abstract

**Introduction:**

*Tetrastigma hemsleyanum* is a folk and rare medicinal plant, and specifically, it is distributed in the south, China. To investigate the cumulative properties of its medicinal components, we examined the effect of UV light on flavonoid content and related enzyme activity changes in *T. hemsleyanum*.

**Methods:**

The leaves and tubers were treated with UV-A, UV-B and UV-C for 1 h, 1L/23D h, 3 h and 3L/21D h (D represents darkness treatment). High-performance liquid chromatography (HPLC) analysis showed that the content of many flavonoids decreased significantly during UV-A treatment, increased after UV-B and UV-C irradiation and accumulated again after darkness treatment.

**Results:**

In the root tubers of the UV-A group, naringin content in the 3L/21D h group (0.069 μg/g) was 16.30 times higher than that of 3 h group (0.0042 μg/g). The rutin content was elevated after UV irradiation but was not detected in the CK group. The test results of the enzyme-linked kit indicated that the activities of many enzymes were higher in the UV-A and UV-B irradiation groups than those in the CK group, but the results were reversed in the UV-C treatment. After darkness treatment, the activities of most enzymes were higher than those with UV irradiation alone; F3’5’H activity in the 3L/21D h group (97.25 U/L) was 1.24 times higher than that in the 3 h group (78.12 U/L) in the UV-A-treated group.

**Discussion:**

The study results suggest that appropriate UV-B and UV-C irradiation, as well as darkness supplementation, had a promotive effect on flavonoids in the leaves and root tubers of *T. hemsleyanum*. Additionally, UV irradiation and darkness treatment enhanced the activity of most enzymes.

## 1 Introduction

*Tetrastigma hemsleyanum* Diels et Gilg, a perennial vine plant belonging to the genus Tetrastigma in the Vitaceae family, is predominantly found in southern China and is utilized in the preparation of traditional Chinese medicine known as “Sanyeqing” (Ji et al., 2020). The entire plant possesses medicinal properties, including heat-clearing and detoxification, wind-dispelling, phlegm-resolving, blood circulation activation, and pain relief (Xu et al., 2016; Ji et al., 2020; Hu et al., 2021). *T. hemsleyanum* leaves are rich in flavonoids (Chen et al., 2022; Zhang et al., 2022), amino acids, terpenoids, cardiac glycosides, and steroids (Sun et al., 2017; Wu et al., 2021). Tubers contain flavonoids, polysaccharides, and terpenes (Feng et al., 2014; Sun et al., 2015; Ru et al., 2019). Notably, flavonoids are the predominant compounds shared between them, demonstrating antiviral (Ding et al., 2019), anti-tumor (Fan et al., 2018; Li et al., 2020b), and anti-inflammatory effects (Wu et al., 2021). *T. hemsleyanum* contains various flavonoid components, such as dihydroflavonoids, flavonoids, and flavonols, including naringenin, rutin, kaempferol, and isoquercetin (Dillon et al., 2017; Li et al., 2020a).

Research has demonstrated that flavonoids are responsive to ultraviolet (UV) radiation and that UV exposure can significantly influence their biosynthesis (Du et al., 2021). For example, UV-A exposure for different durations influences the growth of Chinese kale and phytochemical biosynthesis of flavonoids (Gao et al., 2022). UV-B treatment increases the concentration of various flavonoids and the activity of early stage flavonoid synthetase (Hao et al., 2022). Plant responses to UV-C light have been well-documented (Kostyuk et al., 2008). In a comparison of UV-C treatment of Black Bean Seed Coats with and without UV-C treatment, it was found that UV-C radiation increased flavonoid content in Black Bean Seed Coats (Guajardo-Flores et al., 2014). UV treatment also promoted flavonoid synthase activity in *T. hemsleyanum* (Bai et al., 2022). UV-B induces an increase in RsF3H enzyme activity and accumulation of total flavonoids in the flavonoid biosynthetic pathway (Liu et al., 2013). Notably, flavonoid content is modulated by UV-B duration and varies by species (Neugart and Bumke-Vogt, 2021). Lower levels of UV-B irradiation promote the accumulation of plant flavonoids compared to higher levels of UV-B (Gao et al., 2019). Consequently, we achieved enhanced regulation of trichothecene flavonoids by leveraging the inherent UV tolerance and dark repair mechanisms in plants.

In recent years, the combined analysis of metabolomics and transcriptomics has provided useful insights into the mechanisms of plant regulation by environmental influences, such as in *Ginkgo biloba* (Guo et al., 2021), *Zanthoxylum bungeanum* (Hu et al., 2022), *Hibiseu manihot* L (Zhou et al., 2022), and *T. hemsleyanum* (Xiang et al., 2021). Flavonoids have many contents and types and have a wide range of pharmacological activities in *T. hemsleyanum*; therefore, we used the flavonoid content in the trefoil as the evaluation index. Our previous research demonstrated that flavonoid synthesis was associated with UV irradiation of different durations or wavelengths and that appropriate UV-B and UV-C irradiation of *T. hemsleyanum* could produce a stress response (Bai et al., 2022). Little research has been conducted on *T. hemsleyanum* flavonoids that are UV- and dark-responsive. In this study, we exposed leaves and root tubers to UV-A, UV-B, and UV-C radiation to investigate the effects of various UV exposure times and dark treatments on flavonoid monomer content and associated enzyme activities in the synthesis pathway, and to further explore the possible mechanisms in conjunction with transcriptomics. This establishes the groundwork for additional research on the production and control of flavonoids in *T. hemsleyanum*.

## 2 Materials and methods

### 2.1 Plant materials and treatments

Fresh and healthy three-year-old *T. hemsleyanum* plotted plants from Zhejiang Agriculture and Forestry University (Hangzhou Zhejiang, N 30°15’32’’, E 119°43’23.14’’) were selected as the experimental material. Sixty-five pots of healthy, disease-free, similarly sized *T. hemsleyanum* were selected and randomly divided into 13 groups. With five pots in each treatment group, the UV-A (320 nm–400 nm) radiation intensity was 10 w/m^2^, UV-B (280 nm–320 nm) was 10 w/m^2^ and UV-C (200 nm–280 nm) was 3 w/m^2^, divided into two time periods: light treatment and light treatment + darkness treatment. UV irradiation for 1 h and 3 h (1 h, 3 h), and increased darkness treatment for 23 h and 21 h (1 h + 23 h, 3 h + 21 h). Those who were not exposed to UV light were used as controls and recorded as CK. Samples were collected at the end of the treatment, sealed, and immediately frozen.

### 2.2 Extraction and metabolome determination

Freeze-dried samples were crushed and passed through sieve no. 4. 1.5 mL of 80% ethanol was added to 0.1 g of finely powdered, then sonicated for 30 min. The filtrate was used as the test solution. A total of 23 control solutions were prepared by adding methanol to form a mixed reference solution for each group, which was grouped as follows. Group I: Naringenin, Naringenin, Isorhamnetin, Isorhamnetin-3-*O*-glucoside, Afodoside, Origenin; Group II: Apigenin, Apigenin-7-*O*-glucoside, Asiaticoside, Paclitaxel; Group III: Lignan, Lignan, Isomucoid, Hesperidin; Group IV: Mullein isoflavone glycoside, Quercetin, Isoquercitrin; Group V: Isoorientin, Hypericin, Lenoside, Lenoside; Group IV: Mullein, Quercetin, Isoquercitrin, Rutin, Sageol. The mixed control solutions were stored frozen at 4°C.

Acquity UPLC BEH C18 column (100 mm × 2.1 mm, 1.8 μm) with a constant temperature of 40°C was used. The mobile phase consisted of 0.1% aqueous formic acid (A) and 0.1% aqueous formic acid-acetonitrile (B) in gradient elution at a flow rate (0 min, 5% B; 1 min, 25% B; 3.5 min, 40% B; 4.5 min, 60% B) of 0.6 mL/min. The injected sample volume was 1 μL. Ion source: Turbo V, Ionization mode: ESI-, Volume flow of Curtain Gas (CUR): 30 L/min, IonSpray Voltage (IS): −4,500 V, Volume flow of Ion Source Gas1 (GS1): 55 L/min, Volume flow of Ion Source Gas2 (GS2): 55 L/min. Acquisition method: multiple reaction monitoring mode (MRM) and ionization temperature (TEMP): 550°C. Linearity was analyzed by diluting each of the above-mixed control solutions into seven different mass concentrations of the control solution, and then determining the peak area to analyze the conditions. Linear relationships are presented in **Table 1**.

**Table 1:** Investigation results of a linear relationship.

Compound	Standard curves	R^2^	Linear ranges (μg/mL)
Naringenin	Y = 12,452,147.9 X + 74631.9	0.9992	0.001–0.050
Naringin	Y = 3,972,039.5 X + 64183.6	0.9976	0.010–0.500
Isorhamnetin	Y = 1,476,424.2 X + 2111,521.6	0.9986	0.001–0.050
Isorhamnetin-3-*O*-glucoside	Y = 8,988,517.2 X + 5,992,231.1	0.9982	0.020–1.000
Orientin	Y = 9,071,337.1 X + 33,093.6	0.9996	1.00–100.0
Astragaline	Y = 2,210,508.5 X + 2,507,321.4	0.9986	0.010–0.500
Luteolin	Y = 9,109,434.3 X + 35,359.9	0.9998	0.020–1.000
Luteoloside	Y = 3,168,802.6 X + 166,068.3	0.9994	0.100–5.00
vitexin	Y = 8,542,235.9 X + 18,454,664.6	0.9987	20.0–2000.0
Isoquercitrin	Y = 3,356,305.1 X + 593,847.8	0.9989	0.020–1.000
Rutin	Y = 4,950,860.8 X + 231,551.2	0.9994	0.010–0.500
Eriodictyol	Y = 691,932.1 X + 84,659.5	0.9991	0.010–0.500

### 2.3 Determination of enzyme activity

Determination of PAL activity: Prepared boric acid-borax buffer pH 8.8 was added to 0.5 g of *T. hemsleyanum* leaves or root tubers. A mixture of PVP, EDTA-Na, ascorbic acid, and mercaptoethanol was ground into a homogenate (9 mL) and centrifuged at 4°C for 20 min at 1,000 rpm. The resulting supernatant was used as the crude enzyme. In the control group, 1 mL of phenylalanine solution, 2 mL of distilled water, and 1 mL of boric acid-borax buffer were added sequentially. In the treatment group, 1 mL of phenylalanine solution, 2 mL of distilled water, and 1 mL of crude enzyme were added sequentially. The reaction was terminated by adding 0.2 mL of 6 mol/L hydrochloric acid solution to the tubes after spiking and placing them in a water bath at 30°C for 30 min. Absorbance was measured at 290 nm. CHS, CHI, F3’H, and other enzyme activity assays were performed according to the kit, and enzyme levels were determined by ELISA.

### 2.4 RNA extraction and transcriptome sequencing

Leaf samples were collected from the CK, 1 h, 1 + 23 h, 3 h, and 3 + 21 h groups and snap-frozen in liquid nitrogen. The samples were stored in a refrigerator at −80°C for 24 h and then sent to Nanjing Personal Gene Technology Co. for RNA extraction and transcriptome sequencing. Oligo (dT) was used to enrich the mRNA. An Agilent 2100 Bioanalyzer was used for library quality control. After RNA extraction, purification, and library construction, the libraries were sequenced using next-generation sequencing (NGS) on the Illumina sequencing platform for paired-end (PE) sequencing. Unigene expression levels were calculated for each sample by functional annotation, SSR assay, and filtering out low quality, junctional contamination, and high unknown base N content reads to obtain clean reads and assemble Unigene. For multiple samples, differentially expressed genes were detected between samples according to the requirements, and in-depth clustering and functional enrichment analyses of differentially expressed genes were performed.

### 2.5 Statistical analysis

The data were analyzed using two-way ANOVA analysis and Duncan’s multiple range test (*p <0.05, **p <0.01, ***p <0.001) using Graphpad Statistics. Three independent experiments were performed, and the data points represent the mean ± SEM of three biological replicates. Error bars represent 95% t confidence intervals. Cluster heatmap analysis was performed using the TBtools software. Principal component analysis was performed using Origin 2021.

## 3 Results and discussion

### 3.1 The flavonoids were significantly increased after UV-B treatment in root

**Figure 1:**
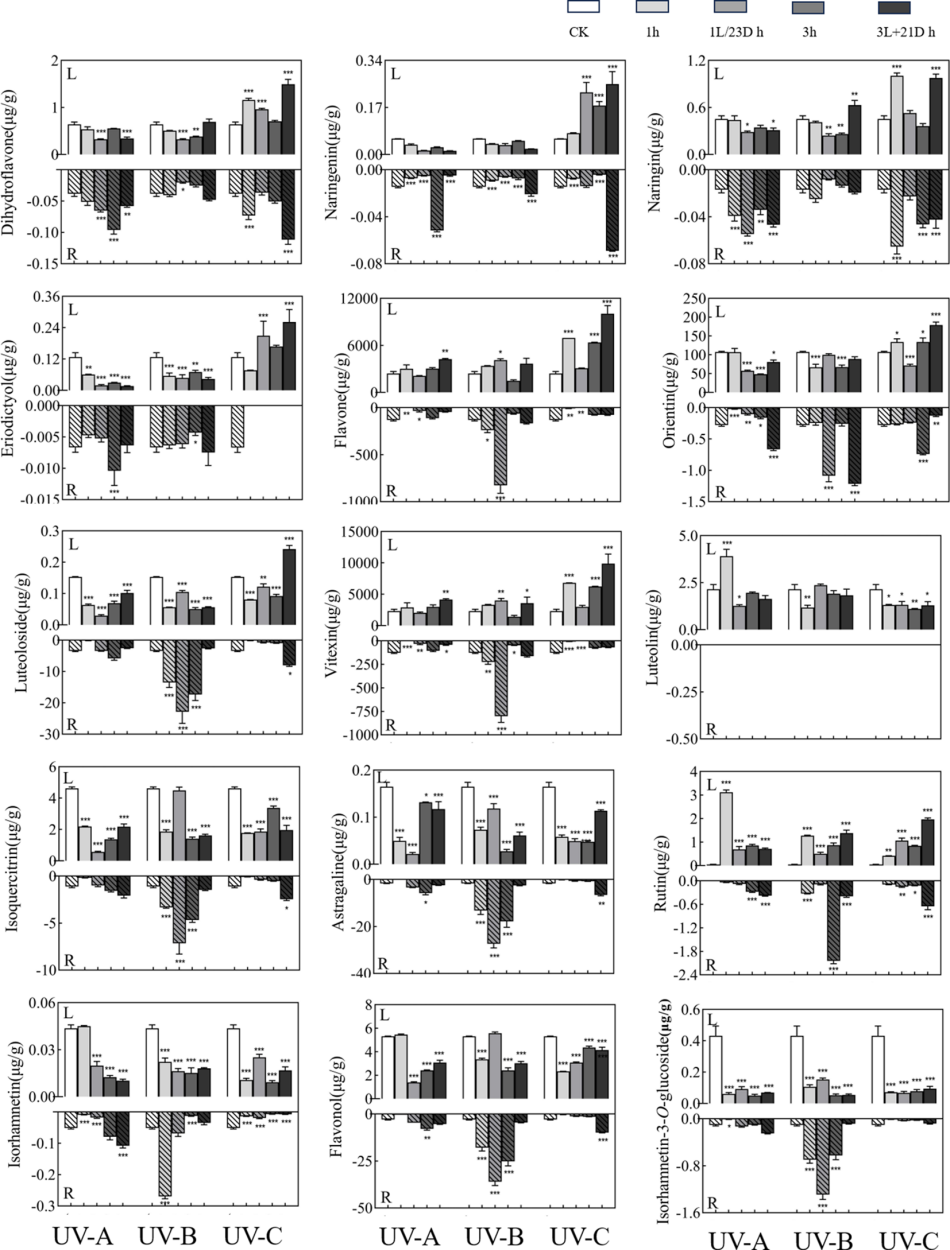
Effect of UV treatments on the content of in monomers. The upper part of the X-axis of each graph indicated the leaves experimental results and the lower part indicated the root tubers experimental results. Three independent experiments were performed, and data points represent the mean ± SEM of three biological replicates. Two-way ANOVA was used for statistical analyses (*p <0.05, **p <0.01, ***p <0.001).

UV-B treatments after 3h of irradiation in darkness treatment increased the content of dihydroflavones in *T. hemsleyanum* roots. The highest content was naringenin (**Figure 1**), which was 16.30 times higher in the 3L/21D h group (0.069 µg/g) than in the 3 h group (0.0042 µg/g). These changes were particularly pronounced in the 1L/23D h group of UV-B, with flavonoids showing trends similar to those of the monomer. The changes were particularly prominent in the 1L/23D h group of UV-B, and the sum of the flavones showed similar trends to those of the monomers. Among them, flavone (238.36 µg/g), orientin (0.25 µg/g), luteoloside (13.47 µg/g), and vitexin (224.64 µg/g) in 1L/23D h group were increased by 3.46 times (825.42 µg/g), 4.43 times (1.09 µg/g), 1.70 times (22.86 µg/g), and 3.57 times (801.47 µg/g) respectively, compared with 1 h group. UV-B treatment also increased flavonol content, and the levels were elevated by the addition of darkness treatment. The flavonoid content reached 35.95 µg/g in the 1L/23D h group, which was 12.19 times greater than CK group (2.95 µg/g) and 2.01 times higher than that in 1 h group (17.87 µg/g). While other monomer concentrations remained comparable across groups, it is noteworthy that rutin which was undetectable in CK was measured at a level of approximately 2.04 µg/g following UV-B treatment for 3 h. However, luteolin was not detected in any of the tubers analyzed.

The content of flavones in the leaves was far higher than that in the root tubers, and the sum of their contents showed an increasing trend after UV irradiation. UV-A and UV-B irradiation had an inhibitory effect on the content of dihydroflavones in the leaves, and the contents were mostly reduced after darkness treatment. In contrast, UV-C treatment significantly increased the content of dihydroflavone, with the highest level in 3L/21D h group (1.49 µg/g), which was 2.33 times higher than CK group (0.64 µg/g), and 2.11 times compared to 3 h group (0.71 µg/g). Meanwhile, darkness treatment after 3 h of irradiation favored reaccumulation. The most dramatic of them was 3L/21D h group (10,052.06 µg/g) of UV-C irradiation, which was 4.16 times more abundant than that of CK group (2,417.88 µg/g), and 1.58 times more than 3 h group (6,369.90 µg/g). In particular, the 3L/21D h group showed a significant increase compared with the CK group. The total flavonol content of the leaves was significantly lower than that of the tubers. In the leaves, the four monomers (astragaline, isorhamnetin-3-*O*-glucoside isoquercitrin, and isorhamnetin) showed similar changes to the sum of flavonols, i.e., the content decreased or changed slightly after treatment. In contrast, the rutin reached 3.11 µg/g after UV-A irradiation for 1 h, which was 67.5 times of the CK group (0.046 µg/g).

### 3.2 insignificant activity cyanoflavone synthase after UV treatment in *T. hemsleyanum*

**Figure 2:**
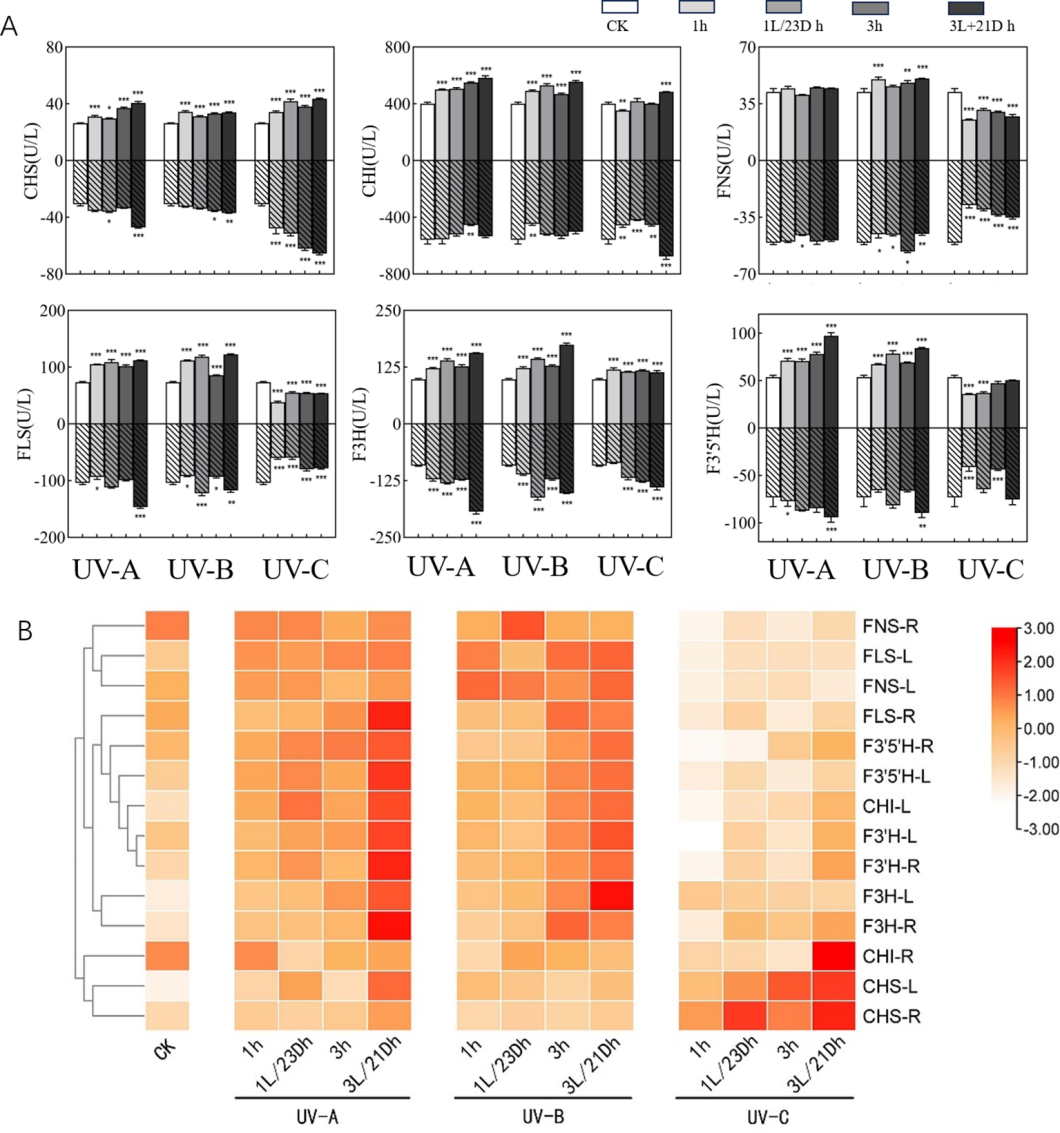
**(A)** Effect of UV-A and UV-C treatments on enzyme activities. The upper part of the X-axis of each graph indicated the UVA experimental results and the lower part indicated the UVC experimental results. Three independent experiments were performed, and data points represent the mean ± SEM of three biological replicates. Two-way ANOVA was used for statistical analyses (*p <0.05, **p <0.01, ***p <0.001). **(B)** Heatmap representing differences in key enzymes activities. Results are visualized using a false-color scale, with red and white indicating an increase and a decrease in the response parameters.

Activities of Chalconesynthase (CHS), chalcone isomerase (CHI), flavonol synthase (FLS), Flavanone 3-hydroxylase (F3H), Flavanone 3’-hydroxylase (F3’H), and Flavanone 3’-5’-hydroxylase (F3’5’H) increased after UV-A and UV-B irradiation in *T. hemsleyanum* leaves. Under UV-C treatment, the activities of both CHS and F3H were elevated, while the activities of the remaining enzymes were lower than those of the CK group, reaching the lowest in the 1 h group, notably F3’H in the leaves (**Figure 2**) [0.52 times (54.52 U/L) lower than that of the CK group (105.36 U/L)]. After darkness treatment, most of the enzyme activities were higher than those during UV irradiation. The greatest increase in F3’5’H by UV-A treatment was observed in the 3L/21D h group (97.25 U/L), which was 1.81 times higher than that in the CK group (53.73 U/L) and 1.24 times more than 3 h group (78.12 U/L). Activity of aforementioned enzymes showed a similar trend in root tubers as in leaves (especially CHI activity in root tubers dropped after UV-A and UV-B treatments and peaked at 3 h (453.89 U/L), which was 0.81-fold lower than that of CK group (557.59 U/L)) and was higher than those in leaves (increase of 16.82%–41.41%, except for F3H and F3’H). CHS was 2.13 times (65.55 U/L) with 3L/21D h treatment more active than CK group (30.74 U/L) in UV-C. This indicated that UV-A and UV-B have a strong effect on promoting enzyme activities in both leaves and root tubers, while UV-C irradiation has an inhibitory effect on enzyme activity but the addition of darkness treatment promoted enzyme activity.

**Figure 3:**
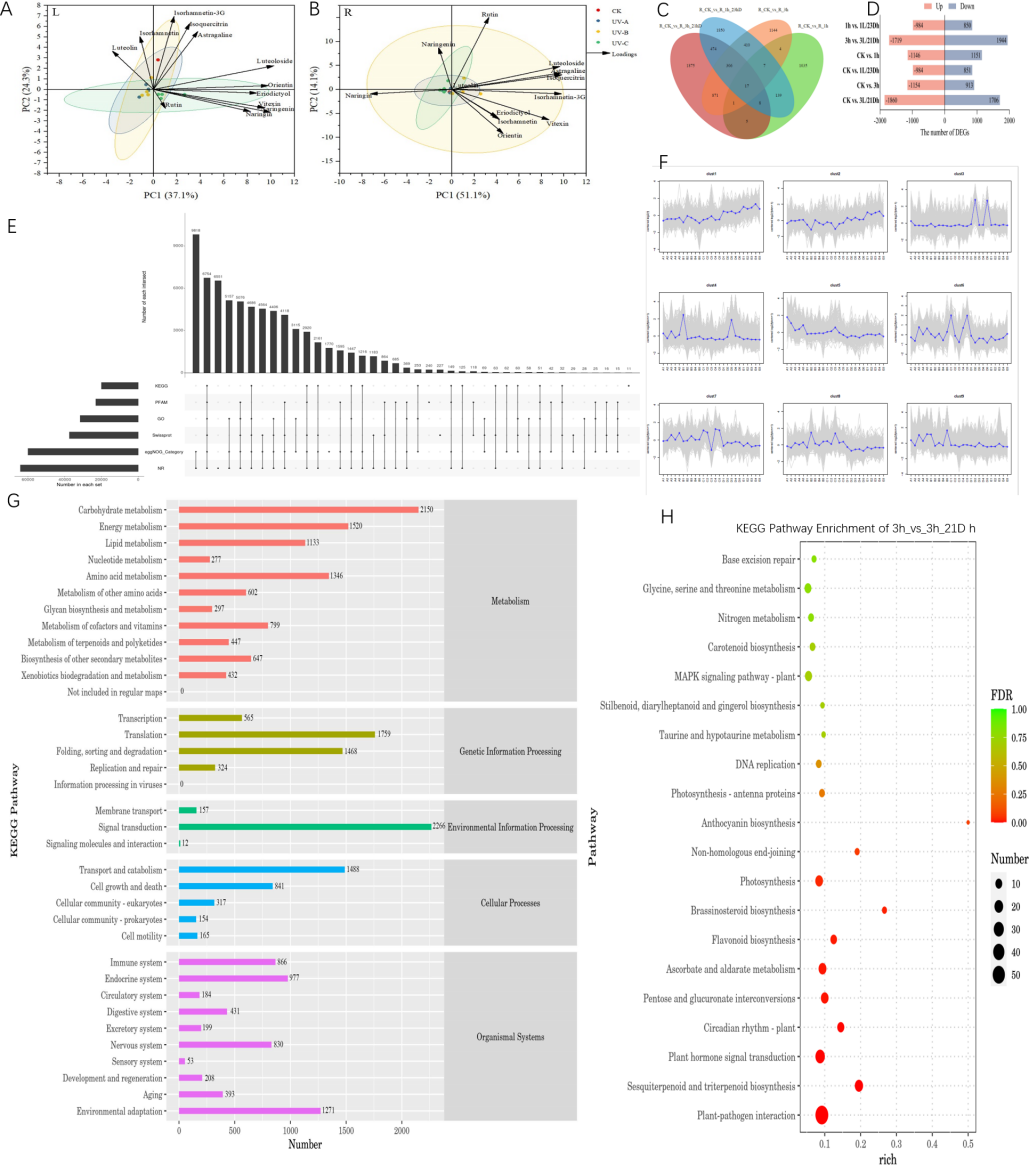
**(A)** was parameter in leaves and **(B)** was parameter in root tubers, Plotted by Origin using the average of three biological replicates of each parameter. **(C)** Venn diagram of the number of DEGs expressed in the different treatment groups. **(D)** The number of DEGs in different comparison groups. **(E)** Unigene Annotated Statistical Results Upset Chart. **(F)** Trend analysis diagram (The gray lines indicate the expression patterns of genes in each cluster, and the blue lines represent the mean of expression in samples across all genes in the cluster). **(G)** KEGG Annotation Statistics. **(H)** KEGG enrichment analysis bubble chart of 3h vs. 3h/21Dh group.

In order to better delineate the effects of UV irradiation and darkness treatment on enzyme activities in leaves and root tubers, we normalized the results and obtained a clustering heatmap. Based on the clustering of the vertical coordinates, the enzymes were split into two major clusters (**Figure 3H**). The first cluster contained CHI-R(R-root), CHS-L (L-leaf), and CHS-R, which were the closest to each other in the measured parameter responses, indicating high activity under UV-C irradiation. The remaining enzymes belonged to the second category, which included 11 enzymes, all of which showed elevated activity upon exposure to UV-A and UV-B, especially after UV-A and darkness treatments. FNS was the most notable enzyme, with lower expression in both the leaves and root tubers than in the CK group.

### 3.3 Combined transcriptome and metabolome analysis of flavonoid-related biosynthetic pathways after UV treatment

To acquire a comprehensive understanding of the response of chemical components to ultraviolet irradiation, a principal component analysis (PCA) model was constructed based on the contents of flavonoids with different durations and darkness treatments. Principal component 1 (PC1) and principal component 2 (PC2) accounted for 37.1% and 24.3% of the total variation, respectively (**Figures 3A, B**). Most flavonoid monomer contents were positively correlated with PC1, indicating that the differences in PC1 were mainly controlled by different durations of UV irradiation and darkness treatment. In the PCA scatter plot, treatments were classified into four groups, namely the distribution of CK, UV-A, UV-B and UV-C treatments. In leaves, the UV-A and UV-B groups were closely distributed, while the CK and UV-C groups were distributed in the other two quadrants. This suggests that the results of UV-C and darkness treatments on leaves differ from those of the UV-A and UV-B treatments. The first two principal components accounted for 65.2% of the total variation in the flavonoid monomer content (PC1, 51.1%; PC2, 14.1%). Most of the monomers were distributed in the UV-B groups, indicating a promising correlation between UV-B treatments and monomer content in root tubers.

Based on the aforementioned results, it was determined that the effect of UV-B irradiation and darkness treatment on *T. hemsleyanum* leaves was more pronounced; thus, further transcriptome analysis was conducted on these leaves. The comparison between groups revealed 96 differentially expressed genes (DEGs), as illustrated in the Venn diagram (**Figure 3C**). Specifically, there were 1,369 DEGs uniquely expressed in CK vs 1 h, 753 DEGs in CK vs 1 + 23 Dh, 2,220 DEGs in CK vs 3 + 21 Dh, and 1,190 DEGs in CK vs 3 h. There were 647 genes associated with the biosynthesis of other secondary metabolites. We employed DESeq for differential analysis of gene expression and screened 15,262 differentially expressed genes with the condition of expression difference multiplicity |log2FoldChange| >1 and significance P-value <0.05. The results indicated both up-regulation and down-regulation among these DEGs; Most DEGs were identified between 3h and 3L/21D h with 3,663, of which 1,719 were upregulated DEGs and 1,944 were downregulated DEGs.

Unigenes were compared to six functional databases for annotation, and finally 64,093 (NR: 50.35%), 31,591 (GO: 24.82%), 20,014 (KEGG: 15.72%), 23,085 (Pfam: 18.14%), 59,954 (eggNOG: 47.10%), and 37,438 (SwissProt: 29.41%) Unigene obtained functional annotations (**Figures 3D–H**). By matching all unigenes to the KEGG database, genes were classified into five branches based on the KEGG metabolic pathways: Metabolism, Genetic Information Processing, Environmental Information Processing, Cellular Processes, and Organismal Systems. Unigenes at levels 1 and 2 of the KEGG database were annotated, and KEGG functional distribution statistics were plotted. Among them, the largest number of genes related to environmental adaptation in Organismal Systems was 1,271. Plant–pathogen interactions (ko04626), sesquiterpenoid and triterpenoid biosynthesis (ko00909), and plant hormone signal transduction (ko04075), among others, were enriched. Notably, the circadian rhythm plant (ko04712), photosynthesis (ko00195), and flavonoid biosynthesis (ko00941) were also included, and the greatest enrichment was observed in anthocyanin biosynthesis (ko00942).

*T. hemsleyanum* was compared with the Plant Transcription Factor Database (PlantTFDB) database to predict the transcription factor and the family to which the transcription factors belong. In total, 10,676 TFs were identified in the five treatment groups, representing 57 different families, among which the Trihelix, bHLH, ERF, MYB, MYB-related, NAC, and WRKY families were transcription factors with a greater number of differential genes. The MYB family comprises 646 transcription factors, while 562 from the NAC family responded to stress conditions alongside 58 for AP2.

**Figure 4:**
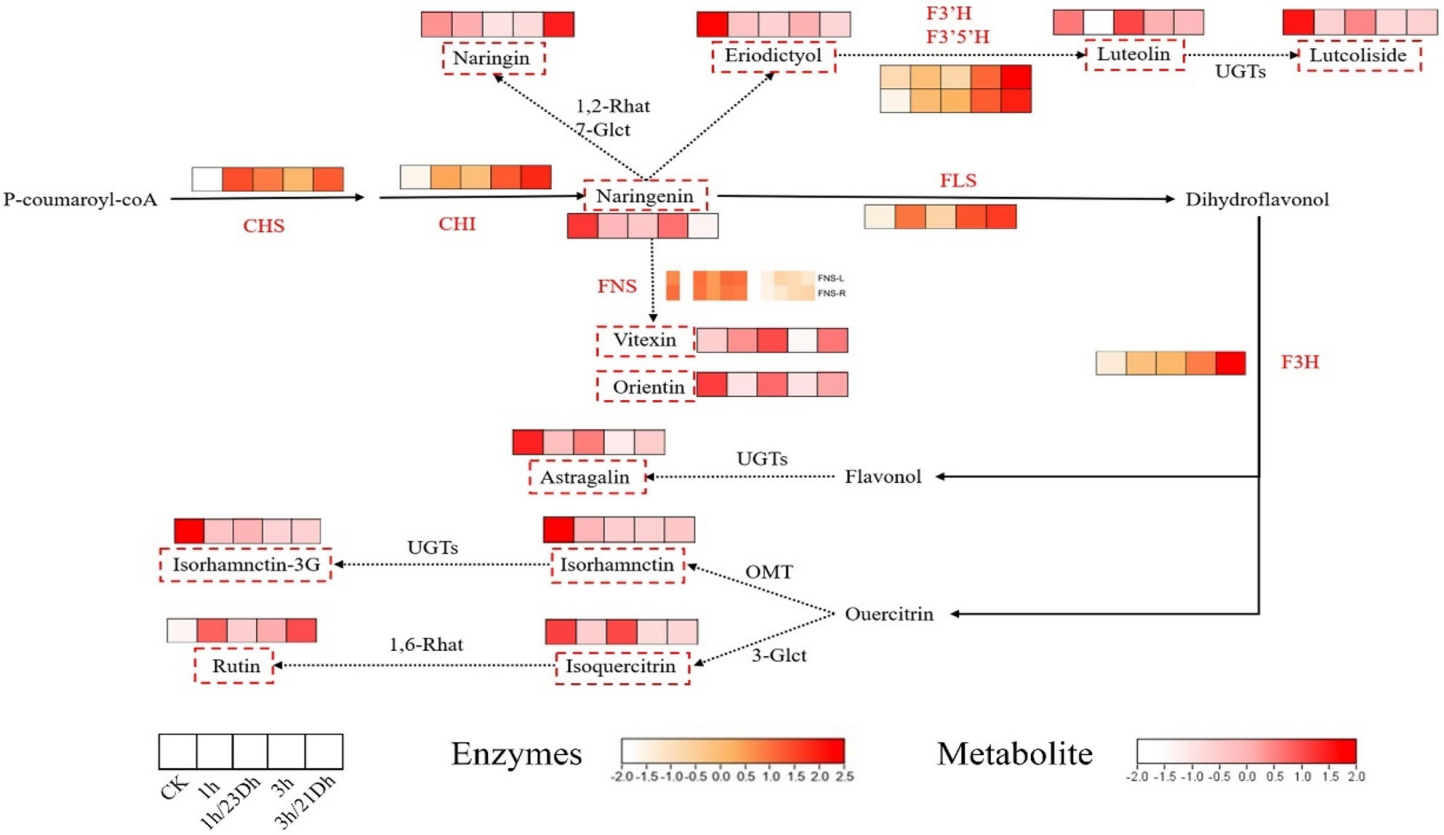
Flavonoid metabolic pathway in leaves. The white and red colors refer to the content level of monomers from low to high. The white and orange colors refer to the expression level of enzymes from low to high. The red dashed box indicates metabolites in this experiment. The red words indicate enzymes in this experiment.

The accumulation of flavonoids and their biosynthetic pathways within *T. hemsleyanum* were constructed based on metabolomic data alongside KEGG pathway analyses, as well as previous research findings (**Figure 4**). In this study, we analyzed the changes in key metabolite content (naringenin, naringin, eriodictyol, orientin, vitexin, luteolin, luteoloside, astragaline, isorhamnetin, isorhamnetin-3-*O*-glucoside, isoquercitrin, and rutin) and key enzyme activities (CHI, CHS, FNS, F3’H, F3’5’H, FLS, and F3H) in flavonoid biosynthesis in *T. hemsleyanum* leaves after UV-B and dark treatments. Among them, naringenin is the key metabolite in flavonoid synthesis, catalyzed by different enzymes, the naringenin produces four branches, one branch produces naringin, the second branch produces eriodictyol, luteolin and luteoloside, the third branch produces orientin and vitexin, and the fourth branch produces astragaline, isorhamnetin, isorhamnetin-3-*O*-glucoside, isoquercitrin and rutin.

## 4 Conclusions

Since the effects of UV radiation on plants depend on exposure time, we evaluated the effects of UV radiation on the content of 15 different flavonoids and activities of seven enzymes in the leaves and root tubers of *T. hemsleyanum* for four different durations and darkness times. The results showed that the effect of UV-B on flavonoids was greater than that of UV-A or UV-C. UV-A irradiation reduced the concentration of monomers in the leaves, whereas UV-B treatment favored the accumulation of monomers in root tubers. Except for CHI in tubers and CHS, all enzymes were more strongly influenced by UV-A regulation. These results suggest that appropriate supplementation with UV-B irradiation and periods of darkness positively influence flavonoid content in both the leaves and root tubers of *T. hemsleyanum.* Concurrently, UV-A treatment appeared to enhance enzyme activity. Our findings have significant implications for both applied and fundamental research concerning *T. hemsleyanum* leaves and root tubers, providing a foundational understanding for regulating the metabolic mechanisms underlying flavonoid biosynthesis through ultraviolet light exposure.

## Data Availability

The datasets presented in this study can be found in online repositories. The names of the repository/repositories and accession number(s) can be found below: https://www.ncbi.nlm.nih.gov/genbank/, PRJNA1137248.
